# Antioxidant therapy, N-acetyl-L-cysteine, effects on pancreatic β cells: therapeutic promise or potential harm?

**DOI:** 10.1210/endocr/bqag114

**Published:** 2026-10-10

**Authors:** Meg Schuurman, Jonathan Nguyen, Malina Barillaro, Nica Borradaile, Rennian Wang

**Affiliations:** Children's Health Research Institute, University of Western Ontario, London, ON N6C 2V5, Canada; Department of Physiology & Pharmacology, University of Western Ontario, London, ON N6A 5C1, Canada; Department of Physiology & Pharmacology, University of Western Ontario, London, ON N6A 5C1, Canada; Children's Health Research Institute, University of Western Ontario, London, ON N6C 2V5, Canada; Department of Physiology & Pharmacology, University of Western Ontario, London, ON N6A 5C1, Canada; Children's Health Research Institute, University of Western Ontario, London, ON N6C 2V5, Canada; Department of Physiology & Pharmacology, University of Western Ontario, London, ON N6A 5C1, Canada; Department of Medicine, University of Western Ontario, London, ON N6A 5C1, Canada

**Keywords:** β cell, insulin secretion, ROS balance, antioxidants, N-acetyl-L-cysteine

## Abstract

N-acetyl-L-cysteine (NAC) is a widely used antioxidant with increasing public interest in its use for treating metabolic disease, yet its effects on pancreatic β cells remain incompletely understood. Most studies focus on NAC's benefits under pathological conditions—such as obesity, high-fat diet exposure, and type 2 diabetes—where oxidative stress impairs β-cell function, identity, and survival. In these contexts, NAC can reduce oxidative damage, preserve insulin secretion, and limit islet fibrosis. However, far fewer studies examine NAC under normal physiological or aging conditions, where moderate reactive oxygen species levels are required for β-cell signaling and glucose-stimulated insulin secretion. Emerging evidence shows that excessive antioxidant exposure during non-pathological states may disrupt redox-dependent pathways, suppress insulin biosynthesis and exocytosis, and contribute to reductive stress. Here, we review the current evidence of NAC's impact on β cells across physiological, aging, and pathological settings. Moreover, we highlight that NAC can be protective or detrimental depending on redox state, metabolic environment, and dosing, which emphasizes the need for careful consideration of antioxidant use in humans.

Redox homeostasis plays a vital role in maintaining pancreatic β-cell function (1, 2). β cells are uniquely sensitive to fluctuations in oxidative states due to their relatively low antioxidant capacity, compared to other tissues (3). The seminal contributions of Lenzen and Jonas established that pancreatic β cells possess unusually weak antioxidant defenses, making them uniquely vulnerable to oxidative stress and metabolic redox imbalances (4-6). Physiological levels of reactive oxygen species (ROS), such as H_2_O_2_, serve essential physiological functions, acting as messengers in insulin synthesis, secretion, and cellular signaling (1, 7). Due to the weak antioxidant defense systems in different subcellular organelles, β cells are particularly vulnerable to the impact of mitochondrial, peroxisomal, and endoplasmic reticulum (ER) stress (8). When excessive ROS accumulation occurs in β cells, it leads to oxidative stress, which in turn contributes to the β-cell dysfunction and apoptosis observed in metabolic diseases, such as type 2 diabetes (T2DM) (9). Conversely, emerging evidence suggests that excessive antioxidant exposure, or reductive stress, may also disrupt redox-dependent signaling pathways (10-13). Among the most widely used antioxidants is N-acetyl-L-cysteine (NAC), a cysteine donor and glutathione (GSH) precursor that has demonstrated both protective and potentially detrimental effects depending on physiological context and dosage (12-14). Antioxidant supplementation has become increasingly common, including among healthy individuals, and understanding the nuanced effects of NAC on β-cell biology and function is of increasing importance. This review will discuss the opposing impacts of NAC, as both a therapeutic and a potential disruptor of intracellular signaling, with a focus on its mechanisms of action, effects on β-cell function, and implications for interventions in health and disease.

## Physiological functions of reactive oxygen species in β cells

Redox signaling refers to the physiological role of ROS in cell signaling and is involved in cell proliferation, differentiation, inflammation, immune responses, and adaptive stress responses. Physiological levels of ROS are regulators of key cell signaling pathways such as PI3K/akt serine/threonine kinase (AKT) and MAPK/ERK signaling cascades (Fig. 1) (15, 16). ROS are formed as a byproduct of cellular metabolism, with the major sources of ROS including mitochondrial respiration and the formation of disulfide bonds during protein folding in the ER (17). Notably, mild stress generates low levels of ROS that trigger mitohormesis, a protective cellular response that signals mitochondria to strengthen and improve overall metabolic flexibility (18). In pancreatic β cells, ROS are a byproduct of physiological cell functions; in fact, ROS generated by the mitochondria are required for glucose-stimulated insulin secretion (GSIS) (1). GSIS-induced physiological levels of ROS production in pancreatic β cells lead to targeted reversible oxidative modifications of proteins (19). A certain level of ER stress, which can be triggered by ROS, is also required for proper insulin folding (2). By applying pharmacological and genetic manipulations, Alfar et al determined that ROS dose-dependently regulate β-cell proliferation *in vivo* and *in vitro*. In particular, reducing ROS levels in β cells blocks their proliferation in response to nutrients (20). Thus, although often regarded as deleterious to tissues, ROS also play essential roles as signaling molecules in biological responses, including in pancreatic β cells.

**Figure 1:**
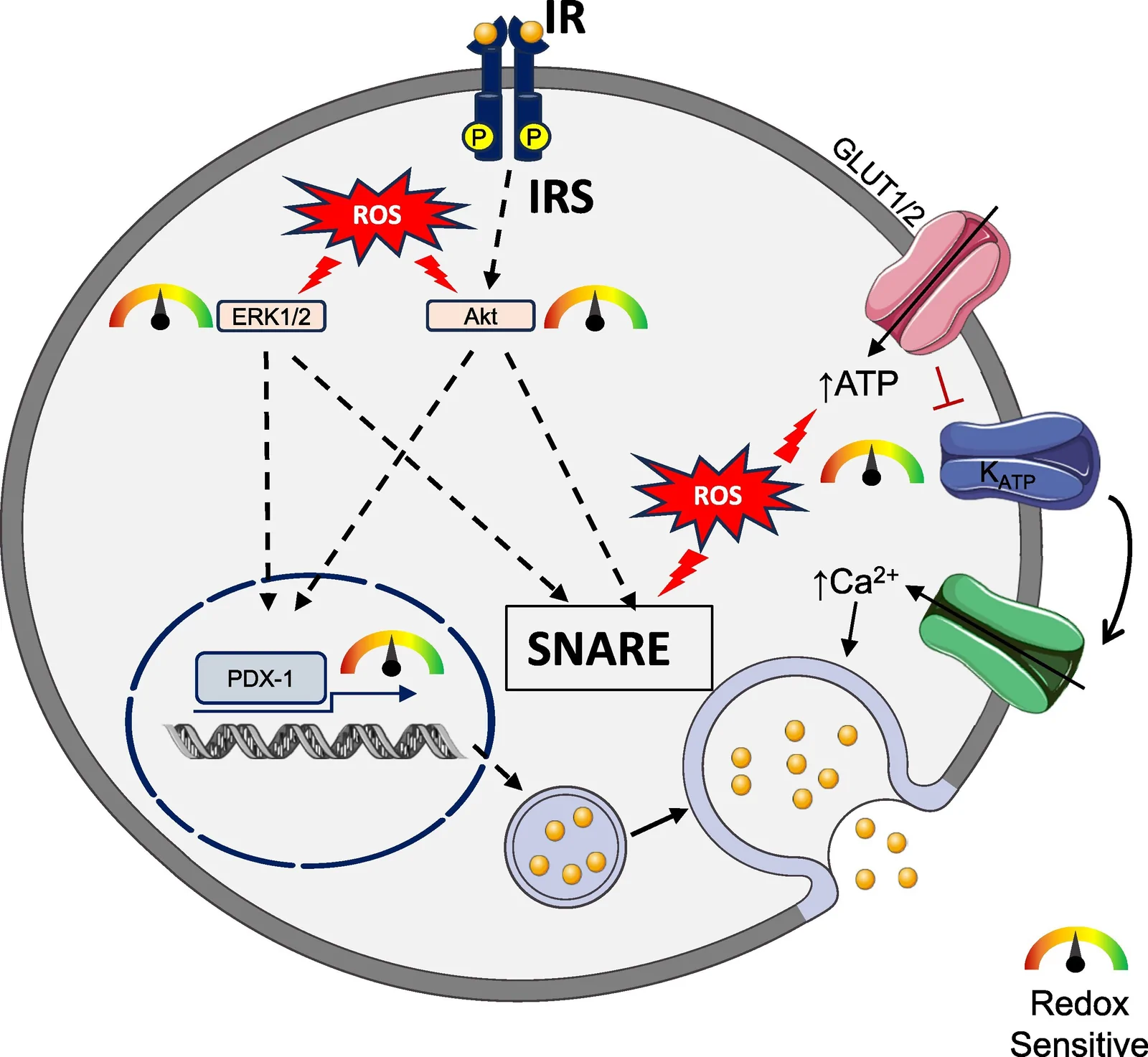
Redox-sensitive pathways in pancreatic β cells. Pancreatic β-cell function is tightly regulated through signaling molecules and transcription factors such as PDX-1, which maintains mature β-cell identity. Major signaling pathways in β cells are insulin receptor signaling, and downstream of that are AKT and ERK pathways. These pathways are redox-sensitive in β cells. Physiological level of ROS signaling is one essential factor for β-cell function and health. ROS are required not only for β-cell intracellular signaling pathways in the regulation of PDX-1 and SNARE signaling, but also for insulin release through the regulation of the SNARE protein complex and K-ATP channel activity. This figure was generated using Servier Medical Art Templates (https://smart.servier.com/).

## Relationships between oxidative stress, obesity, diabetes, and aging

Obesity plays a major role in the progression of T2DM through a variety of mechanisms, one of which is cell and tissue oxidative stress resulting from excess accumulation of ROS. Markers of oxidative stress, 8-isoprostaglandin F2a, and malondialdehyde, were found to be higher in the serum of obese and overweight men compared to lean men (21). In the same men, an inverse relationship between superoxide dismutase (SOD) and intra-abdominal fat area was observed, further connecting obesity with increased oxidative stress (21). Additionally, nitrotyrosine, a marker of oxidative damage not detected in healthy individuals, was found in both male and female T2DM patients’ plasma, and positively correlated with glycemic levels (22). In T2DM progression, obesity is not the only mediator of oxidative stress. Chronic hyperglycemia has been shown to directly contribute to oxidative stress within pancreatic β cells (23).

Compared to other organs, the pancreas has increased susceptibility to oxidative stress due to lower antioxidant enzyme expression. Compared to α cells, pancreatic β cells are extremely susceptible to oxidative stress (24). β cells from human donor islets exhibited a greater decrease in viability and increase in DNA damage in response to oxidative stress compared to α cells, likely due to lower catalase (CAT) and GPx expression (24). In fact, diabetic pancreata have less expression of Cu, Zn-SOD compared to healthy pancreata, amplifying their susceptibility to oxidative stress as demonstrated by the presence of 8-OHdG, a marker of oxidative stress–induced DNA damage (25). Intra-islet pancreatic stellate cells (PaSCs) may also contribute to islet oxidative damage during T2DM through the promotion of islet fibrosis (26). Overall, low β-cell tolerance for oxidative stress combined with the increased presence of oxidative stress in obesity and over-nutrition conditions likely contributes to the β-cell dysfunction observed in T2DM. Understanding the details of how this dysfunction occurs and progresses during T2DM progression may reveal an avenue for diabetic treatments and has been a focus of research.

β cells respond to high glucose levels, but prolonged or chronic high glucose exposure ultimately hinders their function and survival. Similar effects are observed with β-cell oxidative stress. It is undeniable that high and chronic oxidative stress is detrimental to β-cell function. However, at physiological levels, H_2_O_2_ is important for insulin secretion (27). During GSIS studies in both INS-1 cells and isolated mouse islets, an increase in H_2_O_2_ and decreased GSH–GSSH ratio was found to be prevented by NAC and PEG-CAT pretreatment (27). Therefore, it is important to keep in mind that there is an optimal range of oxidative stress, and the goal of treatment should be to maintain the proper balance between antioxidant activity and reactive oxygen species.

Aging has been determined to be a significant risk factor for T2DM, independent of lifestyle and obesity. In humans, there is a positive correlation between glucose intolerance and age (28). In fact, beyond age 30, risk for T2DM development begins to rise exponentially (29). However, aging or elderly individuals are often excluded from clinical studies due to upper age limits and/or multiple comorbidities (30). It is well established that the natural aging process is associated with elevated oxidative stress levels (31). The aging pancreas has elevated levels of mitochondrial oxidative stress, and aging β cells are at increased risk of dysfunction, including reduced insulin secretion, reduced pancreatic and duodenal homeobox (PDX-1) expression, and increased apoptosis (32, 33). Oxidative stress has been heavily implicated in the aging process, and antioxidant therapies have been gaining attention as a preventative for age-related diseases, including T2DM.

## Antioxidant therapies for type 2 diabetes

Diet plays a major role in an individual's antioxidant status, with higher dietary consumption of antioxidants associated with decreased risk of T2DM development (34). There are few studies investigating the preventative effects of antioxidant intake in T2DM in humans; however, existing reports indicate positive results. A large prospective cohort study conducted by Mancini et al examined the association between dietary antioxidant capacity and T2DM risk in 64 223 French women and found that higher levels of total antioxidant capacity were associated with decreased T2DM risk (34). Notably, this positive relationship was linear to a FRAP score of 15 mmol/day, after which a plateau was reached (34). Montonen et al examined men and women from various regions of Finland (35), who did not have T2DM at baseline. Participants were followed for 23 years, with assessments of food consumption and antioxidant intake. The study revealed that increased total intakes of vitamin E and carotenoids, but not vitamin C, were associated with reduced T2DM risk (35). Remarkably, intake of α-tocopherol, γ-tocopherol, δ-tocopherol, and β-tocotrienol were inversely associated with T2DM risk (35). N-acetyl-L-cysteine is not a dietary antioxidant, but has been studied in combination with dietary vitamins. Kaneto et al examined the combination of NAC treatment combined with vitamins C and E in db/db mice (36). N-acetyl-L-cysteine alone provided some improvements in metabolic tests and morphology of islets, but when combined with vitamins C and E, beneficial effects were markedly enhanced (36), suggesting that antioxidant supplementation combined with dietary antioxidants may decrease T2DM risk.

## Antioxidant, N-acetyl-L-cysteine, and its application in clinical practice

N-acetyl-L-cysteine is a potent antioxidant which has received considerable attention for its potential in the management of chronic diseases associated with oxidative stress. There are 3 main proposed mechanisms underlying the antioxidant effects of NAC: (1) oxidant scavenging, (2) disulfide reduction, and (3) GSH replenishment (37, 38). N-acetyl-L-cysteine is a thiol-containing compound that acts as an electron donor to directly remove unwanted disulfide bonds and ROS. After oral administration, NAC reaches the liver, where it is deacetylated to cysteine, a major building block for glutathione (Fig. 2). N-acetyl-L-cysteine has been shown to increase serum GSH levels and, thereby, significantly reduce markers of oxidative stress (39). It has been reported that NAC does not serve as a significant direct chemical scavenger of most biological ROS in pancreatic β cells (40, 41). Instead, NAC undergoes deacetylation in β cells to yield L-cysteine, which is the rate-limiting amino acid for *de novo* synthesis of GSH. Therefore, NAC fuels intracellular enzymatic antioxidant defenses rather than neutralizing radicals on contact (41). The ability of NAC to increase systemic glutathione levels accounts for its indirect antioxidant mechanism and can ameliorate glutathione deficiencies, which are found in patients with T2DM (42).

**Figure 2:**
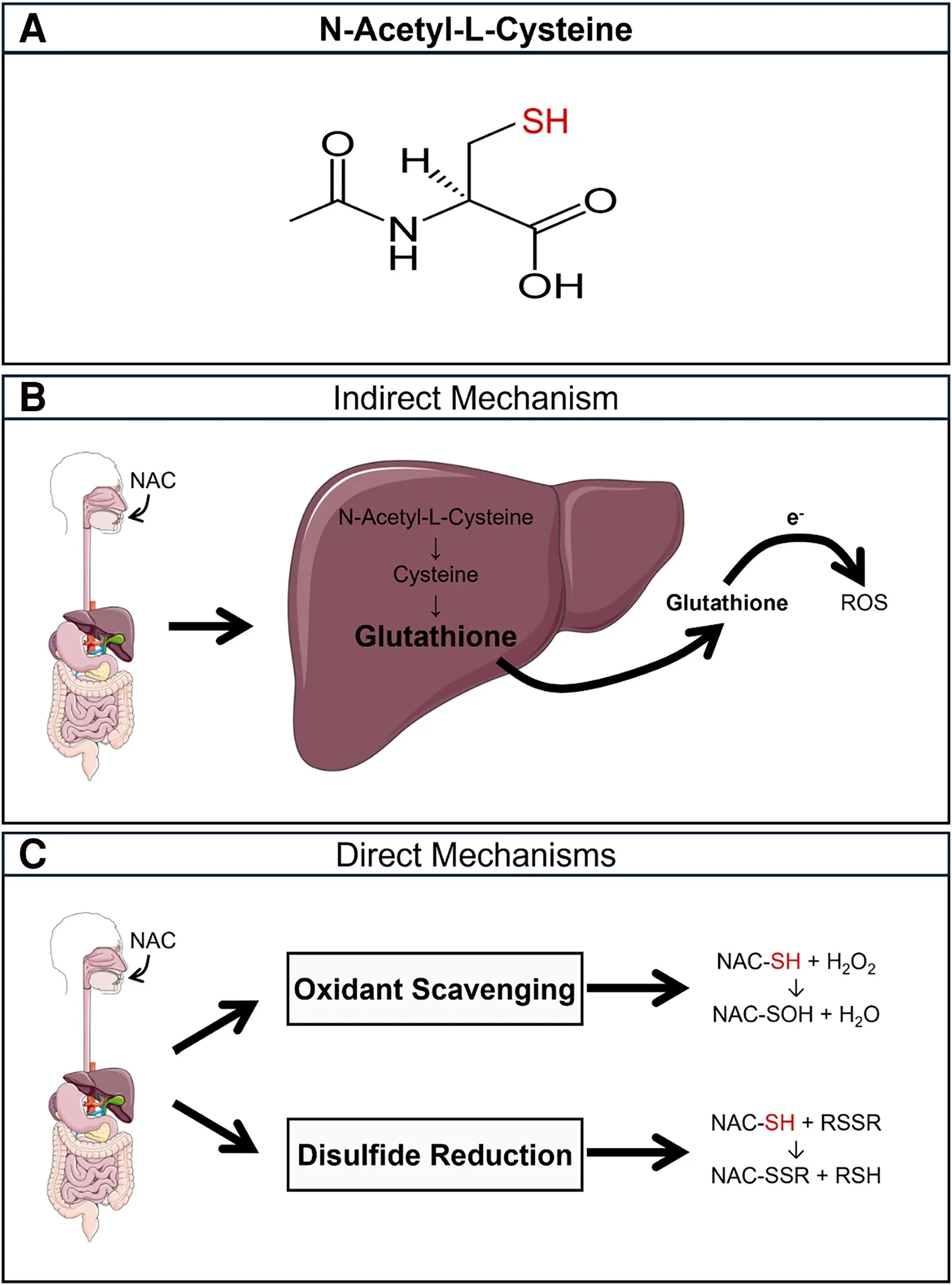
Direct and indirect antioxidative mechanisms of N-acetyl-L-cysteine. (A) N-acetyl-L-cysteine chemical structure. Following oral administration, NAC has antioxidative action through indirect and direct methods. (B) The indirect method is considered the most relevant and predominant in the liver. N-acetyl-L-cysteine is deacetylated to form cysteine—an amino acid that is an essential building block for glutathione synthesis. Glutathione is a potent ROS scavenger and one of the most important molecules in the body for antioxidative defense, which also exists and is of primary importance for antioxidative defense in pancreatic β cells. (C) The thiol group on NAC (-SH) allows it to directly scavenge ROS via electron donation and reduce disulfide bonds. This figure was generated using Servier Medical Art Templates (https://smart.servier.com/).

Beyond its antioxidant capacity, NAC also functions as a mucolytic agent; NAC is currently used in a clinical setting to treat various pathologies such as acetaminophen overdose, chronic obstructive pulmonary disease, polycystic ovarian syndrome, cystic fibrosis, and liver pathologies (43-45).

Historically, NAC has been used successfully in the treatment of various oxidative stress and inflammatory conditions. N-acetyl-L-cysteine can be used in the treatment of lung diseases: chronic obstructive pulmonary disease (COPD) and cystic fibrosis. Standard treatment for COPD uses inhaled corticosteroids and anti-inflammatory agents to prevent exacerbations. However, inhaled corticosteroids can increase oxidative stress and reduce corticosteroid sensitivity. Thiol-containing compounds such as NAC have been investigated as a solo or adjuvant treatment to inhaled corticosteroids to prevent exacerbations (46). In addition to its antioxidant capacity, NAC is also an effective mucolytic agent and is used in the management of cystic fibrosis when administered through inhalation. *In vitro* studies have shown that NAC's disulfide reduction ability may disrupt several bacterial strains in addition to its mucolytic activity. Its glutathione replenishment may also be used in reducing neutrophils and cytokines in the lungs to ameliorate inflammation (44).

Glutathione plays a role in hepatocyte detoxification, protein modification, and apoptosis regulation (47). One of the most well-known clinical uses of NAC is in acetaminophen overdose. In fact, NAC is the only FDA-approved treatment against acetaminophen overdose (48). Acetaminophen overdose results in glutathione depletion and elevated free radicals. N-acetyl-L-cysteine's ability to replenish glutathione and act as an ROS scavenger makes it an ideal antidote for acetaminophen hepatotoxicity (48). There is also evidence to suggest that NAC improves survival rates of those suffering acute liver failure when administered either orally or intravenously (45).

N-acetyl-L-cysteine has been shown to modulate metabolic and endocrine-related diseases such as polycystic ovarian syndrome. N-acetyl-L-cysteine is effective in the improvement of reproductive function and normalizing abnormal hormone levels in patients (49, 50). Key features of metabolic syndrome, low HDL cholesterol, and high fasting blood glucose, were shown to improve with NAC treatment. Overall, NAC is a well-studied antioxidant across a wide range of organ systems.

## N-acetyl-L-cysteine in diabetes therapy

Currently, there is no universally accepted or clinically validated biomarker that reliably predicts whether NAC supplementation alone will be useful for a patient with T2DM (12). However, the antioxidant effects of NAC have been studied in numerous physiological processes and in several diseases, with some promising results in preventing diabetic complications in humans and in mice (51). T2DM patients receiving NAC supplements showed significantly improved mitochondrial fatty acid oxidation, decreased mitochondrial glucose oxidation, lowered plasma free fatty acid levels, and improved insulin sensitivity (52). Prevention of cardiovascular complications related to T2DM, such as critical limb ischemia and atherosclerosis, has been observed alongside decreases in oxidative stress in mouse models of T2DM treated with NAC (53, 54). Finally, NAC treatment prevented T2DM–associated pathological changes in the liver, including hepatocyte degeneration, fibrosis, inflammation, and necrosis (55, 56).

In diet-induced obese C57BL6/N mice, significant improvements in glucose and insulin tolerance and reduced oxidative stress, with preservation of the intra-islet microenvironment, were observed with NAC treatment (57, 58). Optimal NAC dosing (50 mM) and sufficient treatment duration (≥18 weeks) were essential for achieving meaningful improvements in glucose metabolic outcome and β-cell function. N-acetyl-L-cysteine prevented β-cell overcompensation and supported maintenance of β-cell identity in high-fat diet (HFD) fed C57BL6/N mice, demonstrated by normalized β-cell mass, preserved PDX-1 nuclear localization, and reduced intra-islet oxidative stress. Importantly, NAC also diminished intra-islet PaSC activation and collagen deposition, indicating protection against early fibrosis and maintenance of quiescent PaSCs. Collectively, these findings show that the antioxidant and potentially anti-inflammatory actions of NAC preserve β-cell function and the islet niche during diet-induced metabolic stress, supporting further exploration of its potential as an adjuvant therapy for obesity-associated T2DM (57). This study is consistent with previous reports showing the ability of NAC to improve metabolic outcomes in mouse models of T2DM. In KK-Ay mice, NAC improved glucose tolerance and insulin sensitivity in a dose-dependent manner, with most improvements observed at 1200 mg/kg/day. In fact, treatment with this dose resulted in increased pAKT signaling during insulin stimulation (14). In addition to the importance of dose, we and others have highlighted that timing and duration are important factors for effective NAC use during diet-induced obesity (57, 58). In further support of this conclusion, a more recent study demonstrated that aging mice receiving long-term NAC supplementation during an HFD challenge showed improved glucose tolerance and β-cell function, preserved nuclear PDX-1, and enhanced membrane localization of GLUT2 and syntaxin-1A (59). This suggests that NAC mitigates hyperinsulinemia in aging animals by maintaining glucose-sensing and insulin-secretory machinery, while preventing oxidative stress and PaSC activation. Together, these complementary findings strengthen the conclusion that NAC provides robust protection against oxidative stress–induced β-cell dysfunction across both obesity-related and age-associated metabolic challenges.

## N-acetyl-L-cysteine effects on pancreatic β cells in pathological states

Some studies have looked beyond the systemic effects and into the specific changes that NAC may induce in pancreatic β cells. Findings from experiments performed on a rat β-cell line with STZ-induced toxicity showed that NAC treatment increased cell viability, reduced apoptosis, attenuated ROS production, recovered GSH concentrations, improved mitochondrial function, and reduced NOX expression (56, 57). *In vitro*, NAC protected INS-1 β-cells and isolated rat islets from cigarette smoke extract–induced oxidative stress, reducing ROS and apoptosis, improving mitochondrial function, and partially restoring GSIS. Its effects were context-dependent and only partially reversed stress–induced dysfunction (60). N-acetyl-L-cysteine can also prevent changes in transcription factors associated with oxidative stress and glucose toxicity. In the HIT-T15 β-cell line, with exposure to chronic high glucose, late subcultures were found to have significantly lower MAFA protein levels compared to early subcultures (61). N-acetyl-L-cysteine administration, beginning at early subculture, was found to prevent the loss in MAFA protein levels, improve MAFA binding to the insulin promoter, and prevent decreased binding of PDX-1 to the insulin promoter (61). This supports previous work, which found that late subculture loss in insulin mRNA levels was prevented with NAC treatment (62). N-acetyl-L-cysteine has shown beneficial effects on isolated rat islets, where it prevented ribose-induced inhibition of GSIS and a decrease in insulin mRNA and content (23). Recently, we showed that palmitate-induced fatty acid stress in MIN6 cells increased oxidative stress and inhibited cellular ATP production, which resulted in elevated resting insulin secretion with reduced insulin content and alteration to intracellular signaling pathways AKT and ERK1/2. The addition of NAC reduced oxidative stress and restored insulin content; however, NAC further exacerbated reduced ATP production and alterations to AKT and ERK signaling (13).

*In vivo*, NAC reduced oxidative DNA and lipid damage within islets of Zucker diabetic fatty rats, preserving insulin gene transcription and β-cell transcription factor activity, including PDX-1 and MAFA, which maintained GSIS (62). In models of direct β-cell injury, NAC reduced oxidative stress and β-cell apoptosis while preserving β-cell function (63, 64). More recently, in HFD-induced models of metabolic stress, NAC treatment attenuated islet oxidative stress and markers of pancreatic stellate cell activation and fibrosis, indicating effects on both β-cell function and the islet microenvironment (57). We showed that HFD-fed mice administered NAC orally as a preventative treatment showed that it prevented compensatory β-cell mass expansion, preserved the β-cell identity marker PDX-1, and reduced the signal of an oxidative stress biomarker (57). However, we and others identified the critical importance of optimal dosage and duration to maintain redox balance, as systemic overabundance of NAC may cause reductive stress that impairs proper insulin signaling in responsive tissues (12, 57).

## N-acetyl-L-cysteine effects on pancreatic β cells in physiological states

We recently showed that long-term administration of NAC in C57BL6/N mice during aging decreased islet insulin content and secretion, and serum insulin levels (13). Additionally, long-term NAC treatment impaired intracellular signaling pathways in islets, including IR/IRS-1/AKT and ERK1/2 (59). These observations were further supported by our *in vitro* studies in MIN6 cells treated with 10 mM NAC, where we observed reduced AKT signaling and blunted basal insulin secretion (13) as well as reduced cellular ATP content, indicating altered cellular metabolism (13). Similarly, cysteine supplementation has been shown to abolish insulin secretion from β cells via inhibition of PKM2, preventing calcium influx (65). Consistent with our observations of impaired AKT signaling in mouse islets and MIN6 β cells under physiological conditions, NAC also impairs AKT signaling in cultured myotubes and adipocytes (12). Interestingly, AKT activity is highly redox-sensitive. For AKT to be recruited and stabilized at the cell membrane, cysteine residues C60 and C77 undergo reversible oxidation (66). However, under highly oxidative states, AKT can be inhibited through disulfide bond generation between cysteine residues within the activation loop (67). Taken together, these reports suggest that while NAC may protect β cells under oxidative stress, chronic or physiological exposure can impair AKT signaling and reduce insulin secretion.

We recently reported that aging C57B6/N mice receiving long-term NAC supplementation exhibited altered β-cell biology likely through the excess reduction of intracellular ROS. These mice had lower body weight, improved glucose tolerance, and reduced β-cell oxidative stress, PaSC activation, and islet fibrosis. However, these benefits were accompanied by reduced β-cell mass, lower fasting insulin release and islet insulin content, diminished nuclear PDX-1 localization, and decreased expression of insulin exocytosis SNARE proteins. Long-term NAC also suppressed key intracellular signaling pathways—including IR/IRS-1, Akt, ERK1/2, eIF2α, and NF-κB, suggesting that lowering ROS below physiological levels impairs redox-dependent signaling required for maintenance of PDX-1 function, insulin biosynthesis, and secretion (Fig. 3). These observations suggest that tightly regulated intracellular ROS levels are required for proper PDX-1 localization and function. Under physiological conditions, nuclear factor erythroid 2–related factor 2 (Nrf2) serves as a key regulator of cellular redox homeostasis (68). Oxidative stress has been shown to activate both ERK (69, 70) and PI3K/AKT signaling pathways (71, 72), which in turn can promote Nrf2 activation (15, 16). Importantly, β-cell PDX-1 expression and/or activity is enhanced through signaling mediated by Nrf2 (73-75), ERK (76), and PI3K/AKT (77). Collectively, these findings support a model in which intracellular ROS act as signaling mediators that engage Nrf2-, ERK-, and PI3K/AKT-dependent pathways to sustain elevated PDX-1 levels in β cells. Accordingly, prolonged NAC supplementation in aging mice may have reduced intracellular ROS below a threshold necessary for physiological signaling, thereby impairing these pathways and contributing to diminished nuclear localization of PDX-1.

**Figure 3:**
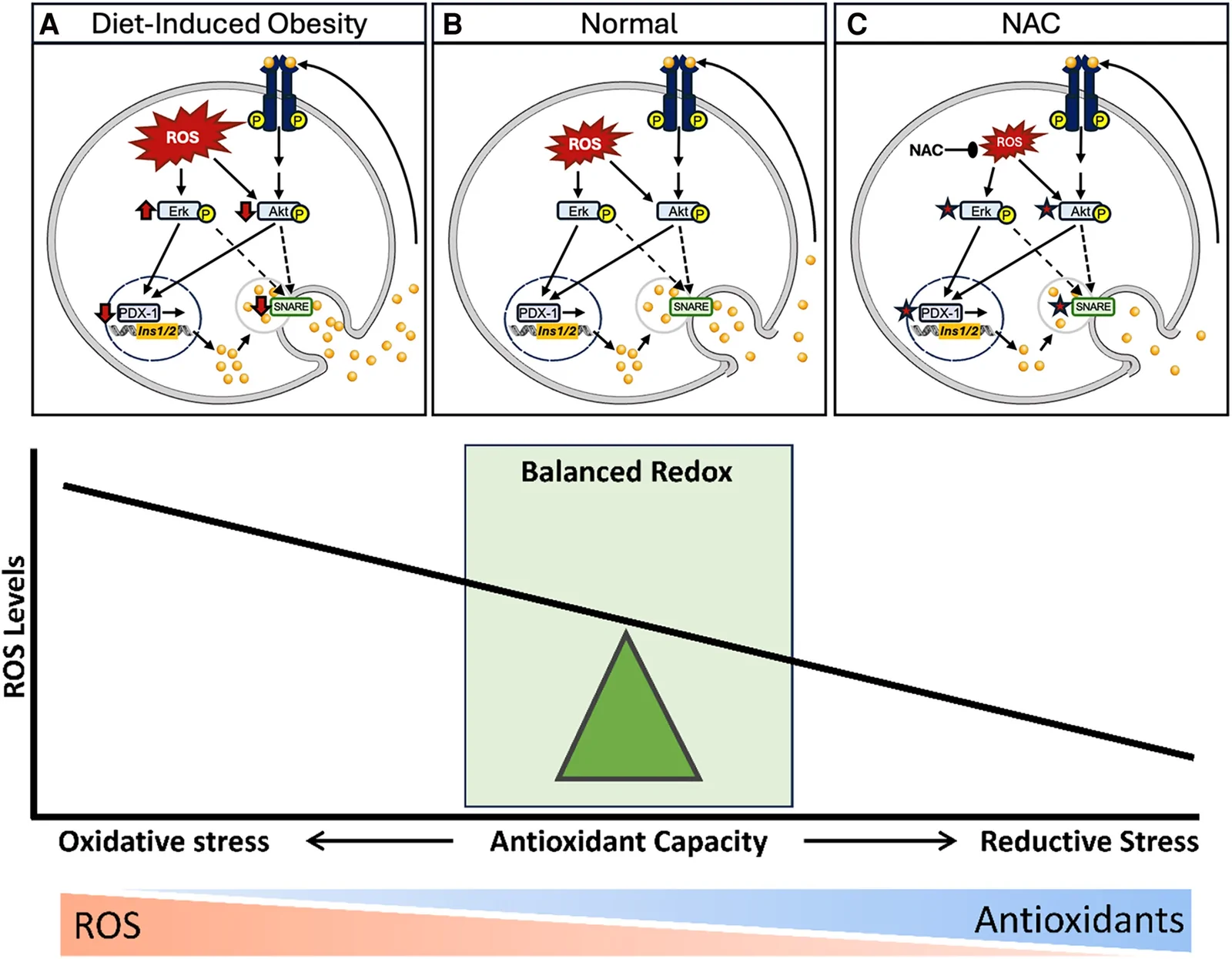
Optimal redox balance is essential for maintaining β-cell function. (A) Pathological levels of ROS, leading to excess oxidative stress, cause down-regulation of Akt phosphorylation, PDX-1, and SNARE protein expression, and resulting in impaired β-cell function. (B) Balanced redox conditions are required for normal physiological functions of β cells, such as maintaining β-cell identity and insulin release. (C) Effects of NAC on β cells are dependent on the conditions during which it is used for treatment. N-acetyl-L-cysteine lowers ROS levels and can impact Erk1/2, Akt, PDX-1, and SNARE protein expression. Long-term NAC supplementation may result in defective insulin secretion, marked by reduced SNARE protein levels and impaired insulin signaling through reduced IR/IRS-1/Akt phosphorylation. This figure was generated using Servier Medical Art Templates (https://smart.servier.com/).

Together, these findings highlight that while NAC effectively reduces pathological oxidative stress, chronic antioxidant exposure under normal aging conditions may induce a state of reductive stress that compromises essential β-cell signaling and insulin-secretory capacity. In line with these findings, we further investigated the impacts of NAC directly on MIN6 β cells. Under physiological culture conditions, NAC altered MIN6 β-cell metabolism and signaling by lowering intracellular ATP and oxidative stress without affecting cell viability, proliferation, or apoptosis. N-acetyl-L-cysteine reduced basal insulin secretion while maintaining normal insulin content, accompanied by a marked reduction in AKT^Ser473^ phosphorylation, indicating that NAC diminished redox-dependent insulin signaling under normal conditions (13). This suggests that while NAC can modulate β-cell metabolic activity, it may simultaneously suppress key intracellular signaling pathways required for physiological insulin secretion, highlighting the importance of maintaining appropriate ROS balance in β-cell function (Fig. 3).

## Summary and perspectives

Although clinical studies assessing the use of NAC in diabetes have produced mixed outcomes, strong preclinical evidence supports its potential to mitigate β-cell oxidative damage, highlighting the need for rigorously designed, comprehensive clinical trials. Importantly, NAC appears most effective as a therapeutic intervention in settings characterized by established oxidative stress, rather than as a preventative strategy. Under physiological conditions, excessive NAC exposure can impair redox-dependent signaling pathways in pancreatic β cells, leading to reduced insulin secretion. These findings underscore the importance of context-dependent redox modulation and suggest that indiscriminate antioxidant supplementation may inadvertently disrupt essential cellular processes. Moving forward, a deeper understanding of tissue-specific redox biology, careful dose optimization, and targeted therapeutic windows will be critical to utilizing antioxidants effectively without compromising physiological signaling or metabolic homeostasis (Fig. 3).

## Data Availability

Data sharing is not applicable to this article as no datasets were generated or analyzed during the current study.

## Search strategies used to obtain information for review

A review of current information was performed through a literature search through PubMed and Google Scholar. Portions of the information regarding NAC on pancreatic β-cell function, highlighting both its protective role under oxidative stress and its potential to impair insulin signaling and secretion under physiological conditions, are based on multiple studies from our laboratory.

## Abbreviations

AKT: akt serine/threonine kinase
CAT: catalase
8-OHdG: 8-hydroxy-2′-deoxyguanosine
ER: endoplasmic reticulum
ERK1/2: extracellular signal–regulated kinases 1/2
FRAP: ferric reducing antioxidant power
GLUT2: glucose transporter 2
GSH: glutathione
GSIS: glucose-stimulated insulin secretion
GPx: glutathione peroxidase
HFD: high-fat diet
MAFA: MAF BZIP transcription factor A
NAC: N-acetyl-L-cysteine
Nrf2: nuclear factor erythroid 2–related factor 2
PaSCs: pancreatic stellate cells
PDX-1: pancreatic and duodenal homeobox
ROS: reactive oxygen species
SNARE: soluble N-ethylmaleimide sensitive-factor attachment protein receptor
SOD: superoxide dismutase
STZ: streptozotocin
